# Effect of Intake of Bifidobacteria and Dietary Fiber on Resting Energy Expenditure: A Randomized, Placebo-Controlled, Double-Blind, Parallel-Group Comparison Study

**DOI:** 10.3390/nu16142345

**Published:** 2024-07-20

**Authors:** Yuhei Baba, Naoki Azuma, Yasuo Saito, Kazuma Takahashi, Risa Matsui, Tsuyoshi Takara

**Affiliations:** 1 Dairy Business Division, Ezaki Glico Co., Ltd., 4-6-5 Utajima, Nishiyodogawa-ku, Osaka 555-8502, Japan; 2 R&D Laboratory, Ezaki Glico Co., Ltd., 4-6-5 Utajima, Nishiyodogawa-ku, Osaka 555-8502, Japan; naoki.azuma@glico.com (N.A.); yasuo.saito@glico.com (Y.S.); kazuma.takahashi@glico.com (K.T.); risa.matsui@glico.com (R.M.); 3 Medical Corporation Seishinkai Takara Clinic, 9F Taisei Bldg., 2-3-2 Higashi-gotanda, Shinagawa-ku, Tokyo 142-0022, Japan; t-takara@takara-clinic.com

**Keywords:** *Bifidobacterium animalis* subsp. *lactis*, probiotics, inulin, visceral fat, metabolic syndrome, gut microbiota, resting energy expenditure, obesity

## Abstract

*Bifidobacterium animalis* subsp. *lactis* GCL2505 in combination with inulin has been shown to have several health benefits, including an improvement in the intestinal microbiota and a reduction in human visceral fat. Previous studies have suggested that the visceral fat reduction of GCL2505 and inulin may be achieved by improving daily energy expenditure. This parallel, placebo-controlled, randomized, double-blind study was conducted to evaluate the effects of GCL2505 and inulin on resting energy expenditure (REE) in overweight or mildly obese Japanese adults (*n* = 44). Participants ingested 1 × 10^10^ colony forming units of GCL2505 and 5.0 g of inulin daily for 4 weeks. REE score at week 4 was set as the primary endpoint. At week 4, the REE score of the GCL2505 and inulin group was significantly higher than that of the placebo group, with a difference of 84.4 kcal/day. In addition, fecal bifidobacteria counts were significantly increased in the GCL2505 and inulin group. Our results indicated that the intake of GCL2505 and inulin improves energy balance, which is known to be a major factor of obesity, by modulating the microbiota in the gut. This is the first report to demonstrate the effects of probiotics and dietary fiber on REE in humans.

## 1. Introduction

Obesity is a state resulting from the excessive accumulation of fat and can have harmful effects on health. The World Health Organization diagnostic criteria defined a body mass index (BMI) of ≥25 kg/m^2^ as overweight and ≥30 kg/m^2^ as obese; in 2022, 43% of adults aged ≥ 18 years worldwide were overweight and 16% were obese [1]. Being overweight or obese has caused an estimated 5 million deaths in 2019 through non-communicable diseases such as cardiovascular disease, diabetes, cancer, neurological diseases, chronic respiratory diseases, and gastrointestinal diseases [2]. Furthermore, if no action is taken, economic losses and healthcare costs due to being overweight or obese are projected to reach $3 trillion annually by 2030 [3], making being overweight or obese one of the greatest public health crises of the 21st century.

Being overweight or obese is primarily caused by an imbalance between energy intake and energy expenditure. Obesity is commonly treated by caloric restriction to lower energy intake as well as exercise to increase energy expenditure. However, with industrialization and urbanization, the widespread use of trains and automobiles has led to reduced amounts of physical activity. Furthermore, it is known that 6 out of 10 obese people live in low- and middle-income countries [4]. Poverty often causes people to select cheap, high-calorie foods [1]. These social factors are some of the reasons for the increase in overweight and obese individuals.

Resting energy expenditure (REE) accounts for the largest portion of the amount of energy a person expends each day (approximately 60%), followed by physical activity (approximately 30%) and diet-induced thermogenesis (DIT; approximately 10%) [5]. It has been reported that the main determinants of DIT heat production are the energy content of the diet and the proportion of protein and alcohol, and it is difficult to control energy expenditure by regulating DIT because energy intake rises as DIT increases [6]. In addition, obese individuals are reported to be less physically active than non-obese individuals [7]. Therefore, REE has attracted attention because efforts can be made to reasonably improve energy consumption. REE consists mainly of respiration, visceral activity, and temperature maintenance. It is known that body temperature is maintained primarily through non-shivering thermogenesis specific to brown adipose tissue (BAT) [8]. A previous study reported that cold stimulation and intake of some capsinoids increased energy expenditure and decreased body fat in participants with low BAT activity [9]. Furthermore, it has been demonstrated not only in experimental animals such as mice but also in humans that BAT dysfunction contributes to obesity [10,11]. These previous studies have suggested that BAT contributes to the elimination of overweight and obesity by increasing energy expenditure through non-shivering thermogenesis.

In addition to cold stimulation and capsinoids, the effects of short-chain fatty acids (SCFAs) on BAT activity and energy expenditure have attracted attention. SCFAs are saturated aliphatic organic acids consisting of one to six carbons, of which acetate (C2), propionate (C3), and butyrate (C4) are the most abundant (≥95%). The relationship between SCFAs and BAT activity has been studied, and animal studies have reported that the administration of butyric acid activates BAT and improves energy expenditure [12], while administration of acetic acid enhances the expression of genes related to BAT function and increases oxygen consumption, an indicator of energy expenditure [13]. Furthermore, human studies have shown that the infusion of a mixture of SCFAs into the intestine improves lipid oxidation and REE compared with the placebo [14]. These previous studies have suggested a possible effect of SCFAs on energy expenditure via the activation of BAT; the most reasonable way to synthesize SCFAs is fermentation in the body by intestinal bacteria [15]. Non-digestible carbohydrates (dietary fiber) are fermented to produce energy for bacterial growth, and SCFAs are produced as the main end product [16]. Therefore, probiotics, which produce SCFAs in the gut, prebiotics, which are capitalized by intestinal bacteria, and synbiotics, which are a complex of probiotics and prebiotics, are suitable materials for increasing the level of SCFAs in the gut. Previous animal studies have shown that probiotics and prebiotics support the BAT-mediated enhancement of REE. The combined administration of the probiotic strain *Bifidobacterium adolescentis* 2016_7_2 and a high-fat diet leads to a decrease in the respiratory quotient (RQ) and an increase in the expression of Ucp-1 in BAT [17], and the intake of the prebiotic caffeoylquinic acid improves energy expenditure with the help of the microbiota [18]. In humans, however, there have been no reports of improved energy metabolism by probiotics, prebiotics, or synbiotics, although there are reports of enhanced fat oxidation with the administration of oligopeptides [19] and 24 g of inulin [20].

*Bifidobacterium animalis* subsp. *lactis* GCL2505, commercially named “BifiX” in Japan, is a probiotic strain that grows in the gut that passes through and was originally isolated from the feces of healthy adults [21,22]. Previous animal experiments have revealed that SCFAs produced in the intestine by GCL2505 have anti-metabolic syndrome effects such as improving glucose tolerance and suppressing visceral fat accumulation [23] and affect host metabolic homeostasis, including the enhancement of glucose tolerance and suppression of body fat accumulation, via G protein-coupled receptor 43, which is also known as the SCFA receptor [24]. Clinical studies have reported that the intake of GCL2505 was associated with improved cognitive function [25] and vascular endothelial function [26] when administered in combination with inulin [27], a typical prebiotic material. Furthermore, we recently conducted a clinical trial to evaluate the efficacy of GCL2505 and inulin on obesity [28]. In healthy adult men and women with a BMI of ≥23 kg/m^2^ and <30, 12 weeks of GCL2505 and inulin intake significantly decreased visceral and total fat area, increased the total number of bifidobacteria, and decreased the levels of several lipid markers. Therefore, it was suggested that the combined intake of GCL2505 and inulin improves the intestinal environment and reduces abdominal fat related to the SCFA-mediated pathway. Because GCL2505 can proliferate in the gut, it may contribute to the increase in SCFA levels in the gut, thereby exerting anti-obesity effects.

In other words, the effect on visceral fat caused by the combined intake of GCL2505 (a high producer of SCFAs) and inulin is thought to be due to the increased presence of SCFAs in the gut, but the mechanism of action has not been clarified. From the previous studies, it was considered that the visceral-fat-reduction effect of GCL2505 and inulin may be achieved by improving daily energy expenditure. Therefore, the present study conducted a parallel, placebo-controlled, randomized, double-blind study to evaluate the effects of intake of the synbiotics GCL2505 and inulin on REE in overweight or mildly obese Japanese adults.

## 2. Materials and Methods

### 2.1. Participants

This study was approved by the Ethics Committee of Medical Corporation Seishinkai Takara Clinic on 20 September 2023 (approval number: 2309-00178-0078-3BTC). Written informed consent was obtained from all participants in accordance with the Declaration of Helsinki. Participants were Japanese men and women between the ages of 25 and 61 years at the time of consent who satisfied the inclusion criteria, did not satisfy any of the exclusion criteria, and were deemed eligible by the study investigator. The inclusion criteria were as follows: (1) in good health, (2) BMI between 25 kg/m^2^ and 30 kg/m^2^, (3) body fat percentage of at least 15% in men and 25% in women, and (4) the top 40 participants with the lowest resting energy metabolism among those satisfying selection criteria (1) through (3) and not satisfying the exclusion criteria. The exclusion criteria were as follows: (1) undergoing treatment for or a history of malignant tumor, heart failure, or myocardial infarction, (2) having a pacemaker or implantable cardioverter-defibrillator, (3) undergoing treatment for arrhythmia, liver damage, kidney damage, cerebrovascular disease, rheumatism, diabetes, dyslipidemia, hypertension, or other chronic diseases, (4) consuming foods for specified health uses or foods with functional claims, (5) taking pharmaceuticals (including herbal medicines) or supplements, (6) having allergies (to pharmaceuticals or food related to the tested food), (7) pregnant, lactating, or intending to become pregnant during the study period, (8) participation in other clinical trials during the 28 days prior to the date of consent, (9) took antibiotics during the 28 days prior to the date of consent; and (10) deemed ineligible by the principal investigator.

### 2.2. Management of Participants

Participants were managed as follows: (1) During the study period, the ingestion or non-ingestion of test drinks and the occurrence of menstruation (for women only) were recorded daily in a logbook provided by the contract research institute. (2) The physical condition of the study participants was ascertained by interview at the time of their visit to the hospital. (3) Dietary intake was examined 3 and 2 days before each test day. A Calorie and Nutrition Diary (CAND) was used for the dietary survey [29]. The participants were asked to submit their CAND at the time of each examination visit. (4) For breakfast and lunch on the day before each test, participants consumed the specified prescribed diet. Participants spent the evening at their designated accommodation facility and consumed the prescribed dinner no later than 12 h before the start of the test the next day. Participants were asked to avoid eating and drinking anything other than the prescribed diet and were only allowed to drink water. (5) Participants were asked to ensure compliance with the following points during study participation: (a) from the date of obtaining consent to participate in the study until the final test (4 weeks post-test), avoid binge eating and drinking and maintain their previous lifestyle; (b) if any change in physical condition occurs during the study period, immediately contact the sponsoring clinical research organization and ask for instructions on what to do next; (c) consume the test drinks according to the prescribed dosage and administration, at an intake rate of at least 80%; (d) during the test period, avoid, to the extent possible, consuming foods for specified health use, foods with functional claims, fermented foods such as yogurt, kimchi, and natto, and other foods/beverages with possible functional properties; (e) avoid alcohol consumption and excessive exercise from 3 days before each examination until the end of the examination on the same day; (f) refrain from consuming caffeine-rich beverages (energy drinks, coffee, etc.) for 3 days before the test, and avoid consuming caffeine-containing beverages on the day before the test.

### 2.3. Test Foods

The test foods were a dairy drink containing inulin (Orafti GR; Beneo GmbH, Mannheim, Germany) and GCL2505 (active drink) or a placebo. The active drink was made by diluting the fermented dairy drink in which the bifidobacteria count was measured with a non-fermented dairy drink containing the same ingredients to stabilize the bifidobacteria count. The active drink contained 5.0 g of inulin and 1 × 10^10^ colony-forming units of GCL2505 per 100 g. The placebo was prepared with the same ingredients as the active drink, with the addition of food-grade acetic acid and lactic acid to adjust flavor and pH; the basic ingredients were skim milk powder, fructose dextrose, sucrose, yeast extract, acidifier, stabilizer, and flavoring. The nutritional details of the test foods are shown in Table 1.

### 2.4. Experimental Design

**Figure 1:**
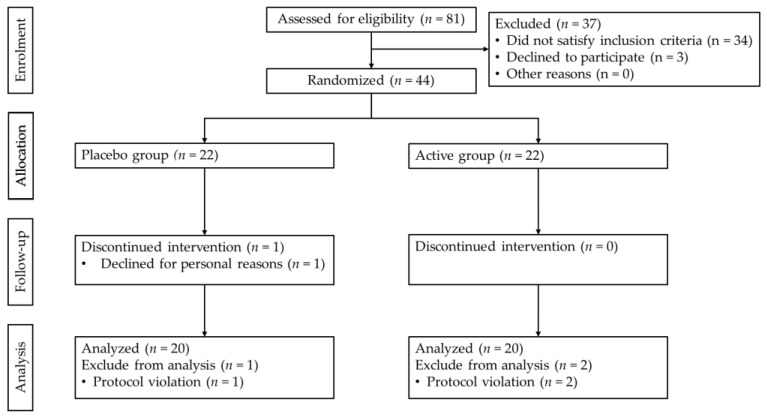
Flowchart of participant selection.

The study was a randomized, placebo-controlled, double-blind, parallel-group study. Participants who satisfied the eligibility criteria at the time of the screening test were assigned by the allocation manager to either the active or placebo group at a 1:1 ratio, using an allocation table generated by the open-source software program R (ver. 4.2.1); the algorithm used block random allocation with a random block size of seven. For the sample size, the final target number of participants was set at 20, based on our previous study of resting energy expenditure with GCL2505 and inulin (UMIN000050836, unpublished). The participant selection process is shown in Figure 1. In this study, 81 participants were screened. After screening, 44 participants were eligible; 22 were assigned to the active group and 22 were assigned to the placebo group. The doses of GCL2505 and inulin were determined based on previous studies, respectively [28,30]. Participants in the active and placebo groups consumed 100 g of test foods once daily for 4 weeks. Both the participants and observers were blinded to group allocation for the duration of the study. Double blinding was accomplished by labeling the test foods with an identification number only. The identification numbers of the active and placebo drinks were kept strictly confidential and were not disclosed until the allocation manager sent out the allocation list after the study was completed. The allocation manager generated the allocation order based on the identification numbers of the test foods provided and created an allocation list and an emergency key. The emergency key was sealed in an envelope for each study participant, and the envelope was stamped with an allotment seal and sealed. After the study was completed and the data were fixed, the allocation manager confirmed that the allocation list and emergency key had not been opened, and the identification numbers of the test foods were revealed. The primary outcome was REE at week 4. Secondary outcomes included REE at week 2, RQ, carbohydrate oxidation, fat oxidation, body weight, BMI, body fat percentage, and muscle mass at weeks 2 and 4, as well as fecal SCFAs (formic acid, acetic acid, lactic acid, propionic acid, n-butyric acid, iso-butyric acid, succinic acid, n-valeric acid, and iso-valeric acid) and the number of bifidobacteria in feces at week 4. The study was conducted at the Medical Corporation Seishinkai Takara Clinic (Tokyo, Japan) from October to December 2023 by Orthomedico Inc., a contract research organization (Tokyo, Japan), and was registered with the University Hospital Medical Information Network Clinical Trials Registry as UMIN000052435. This article conforms to the Consolidated Standards of Reporting Trials (CONSORT) 2010 guidelines (Supplementary Materials, Table S1).

### 2.5. Indirect Calorimetry

Participants’ oxygen uptake (VO_2_) and carbon dioxide production (VCO_2_) were measured using a respiratory gas analyzer (AE310S; Minato Medical Science, Osaka, Japan) in the morning of the test day. Participants were instructed to rest in their assigned accommodations from the evening before the test and to consume their assigned dinner in the accommodations at least 12 h before the start of the test the next day. Measurements were taken in a resting sitting position at a comfortable room temperature; VO_2_ and VCO_2_ were recorded continuously for 15 min. REE and RQ were calculated by the following equations:

REE (kcal/day) = [3.9 × VO_2_ (mL/min) + 1.1 × VCO_2_ (mL/min)] × 1.44 (1)

RQ = VCO_2_/VO_2_ (2)

### 2.6. Anthropometric Measurements and Body Composition

Body weight and height were measured in units of 0.1 kg and 0.1 cm, respectively, with the participant standing. BMI was calculated by dividing weight (kg) by the square of height (m).

### 2.7. Fecal Samples

Fecal samples were submitted on weeks 0 and 4. Fecal samples were handled according to previously described procedures [28] and promptly transported to the Kyoto Institute of Nutrition and Pathology (Kyoto, Japan) by refrigerated transport at temperatures below −15 °C.

### 2.8. Fecal Short-Chain Fatty Acids

Concentrations of SCFAs in feces were determined using ion-exclusion high-performance liquid chromatography (HPLC) according to the procedure of Morishima et al. [31]. Specifically, 0.3 g of feces was placed into a 1.5 mL microtube. To a suspension consisting of feces and 0.6 mL distilled water, 0.09 mL of 12% perchloric acid was added and allowed to stand on ice for 3 min after suspension. Samples were then centrifuged (15,000× *g*, 10 min, 4 °C), and the collected supernatant was filtered through a 0.45 µm COSMONICE^®^ Filter W (water-based: Nacalai Tesque, Inc., Kyoto, Japan) before being subjected to analysis. An LC-10ADVP pump, CDD-10A VP conductometer, Shim-Pack SCR-102(H) Column (8.0 mm × 30 cm × 2 columns), and CTO-20AC column heater module (all manufactured by Shimadzu Corporation, Kyoto, Japan) were used to measure the analytical concentrations of SCFAs. Distilled water for HPLC (Fujifilm Wako Pure Chemical Corporation, Osaka, Japan) containing 5 mM *p*-toluenesulfonic acid was prepared as the mobile phase and filtered through a 0.45 µm cellulose acetate membrane filter (Toyo Roshi Kaisha, Ltd., Tokyo, Japan) for use in the measurements. The post-column pH buffering solution was distilled water with 5 mM *p*-toluenesulfonic acid, 20 mM Bis-Tris, and 100 µM EDTA (free acid) added. Mobile phase and pH buffering solution were supplied at a flow rate of 0.8 mL/min each. The column temperature was set at 45 °C. Components were identified by the Kyoto Institute of Nutrition and Pathology (Kyoto, Japan), using a CBM-20A data module (Shimadzu Corporation, Kyoto, Japan).

### 2.9. Fecal DNA Extraction

Bacterial DNA was extracted from fecal samples, according to the procedure of Tourlousse et al. [32]. Specifically, 0.2 g of fecal sample, 700 µL of FE1 buffer, and 10 µL of RNase were added to a tube containing beads, and a bead-beating homogenizer (FastPrep-24; MP Biomedicals, Irvine, CA, USA) was run at 6 m/s for 1 min to destroy the cells. This process was repeated three times, during which the samples were held at room temperature for 5 min. The samples were then centrifuged at 12,000× *g* for 15 min with 90 µL of FE2 buffer added. The supernatant (up to 500 µL) was collected and mixed with FB buffer and isopropanol, each at 0.4× the volume of the supernatant obtained. Finally, the sample was loaded onto a spin column and washed according to the manufacturer’s instructions. The purified DNA was eluted from the column by 50 µL of Tris-EDTA buffer (pH 8.0).

### 2.10. Fecal Bifidobacteria

Real-time polymerase chain reaction (PCR) was performed with reference to Tanaka et al., using genus-specific primers capable of detecting *Bifidobacterium* spp., including GCL2505 [33]. The primer sequences were as follows: *Bifidobacterium* spp. sense primer, 5′-GATTCTGGCTCAGGATGAACGC-3′; *Bifidobacterium* spp. antisense primer, 5′-CTGATAGGACGCGACCCCAT-3′. Each PCR reaction mixture contained 20 pmol of each primer, 5 µL of SYBR^®^ premix Ex taq (Takara Bio, Kusatsu, Japan), and 1 µL of DNA solution. This procedure was performed by the Kyoto Institute of Nutrition and Pathology (Kyoto, Japan).

### 2.11. Statistical Analysis

All measurement data are presented as means and standard deviations (SD) or 95% confidence intervals (CIs). All statistical analyses were performed using IBM^®^ SPSS^®^ Statistics 23 (IBM Corp., Armonk, NY, USA). *p*-value < 0.05 was used as the threshold for determining significant differences. Missing data were treated as missing values and no proxy values were used. Unpaired *t*-tests were used to assess baseline at study entry between participants in both groups and dietary bias during the study period. The participants’ indirect calorimetry and body parameters during the study period were compared between groups, using a mixed-effects model for repeated measures, based on the restricted maximum likelihood method. The mean structure of models other than BMI assumed a fully unstructured variance–covariance matrix for error terms, including time, group (active or placebo), sex, baseline values, baseline BMI values, and interactions between time and group, and between baseline values and time. The mean structure of models for BMI included baseline value, time point, group (active or placebo), sex, interaction between time point and group, and interaction between baseline value and group, and assumed a fully unstructured variance–covariance matrix in the error term. Satterthwaite’s method was used to estimate the degrees of freedom. The difference between groups at each time point was calculated as the difference in the marginal estimated means. Fecal SCFAs concentrations were statistically analyzed by covariate analysis adjusted for baseline (week 0). Fecal bifidobacteria were compared within each group by paired *t*-test and between the two groups by a covariate analysis adjusted for the baseline (week 0).

## 3. Results

### 3.1. Subjects (Analysis Target Population)

At the start of the study, a significant difference in plasma glucose values between the active and placebo groups was observed but was deemed acceptable because it was within the reference range. There were no differences in the baseline characteristics of other participant data between the two groups (Table 2). By the end of the study, one participant from the placebo group withdrew for personal reasons. After the completion of the entire study, three participants whose consumption rate of the test food was less than 80% were excluded according to study participant management criteria (5)-(c) (*n* = 1 from the placebo group and *n* = 2 from the active). Finally, 40 participants were analyzed, with 20 in the placebo group and 20 in the active group. There were no reported harms or unintended effects in either group.

### 3.2. Dietary Composition

Nutrients ingested by the participants were calculated from the food records for 2 days before REE measurement (Table 3). No statistically significant differences were observed between the two groups in energy, protein, fat, carbohydrate, and dietary fiber. Participants were required to eat the designated dinner at the provided accommodations no later than 12 h before the start of the next day’s test, thereby ensuring that the content of the meal the day before the REE measurement did not affect the test results. Accordingly, it was concluded that dietary content did not affect the results of this study.

### 3.3. Indirect Calorimetry

The REE score at week 4 (the primary endpoint) of the active group (1376.5 ± 272.8 kcal/day) was greater than that of the placebo group (1303.2 ± 188.1 kcal/day), and a significant difference was confirmed (*p* = 0.042 by repeated measurements analysis using a linear mixed model). In addition, the REE score in the active group at week 2 (1435.9 ± 195.2 kcal/day) was also statistically higher than in the placebo group (1345.5 ± 231.6 kcal/day) (*p* = 0.002 by repeated measurements analysis using a linear mixed model). In contrast, no significant differences were observed between the two groups in RQ, carbohydrate oxidation, or lipid oxidation (Table 4).

### 3.4. Fecal Bifidobacteria

**Figure 2:**
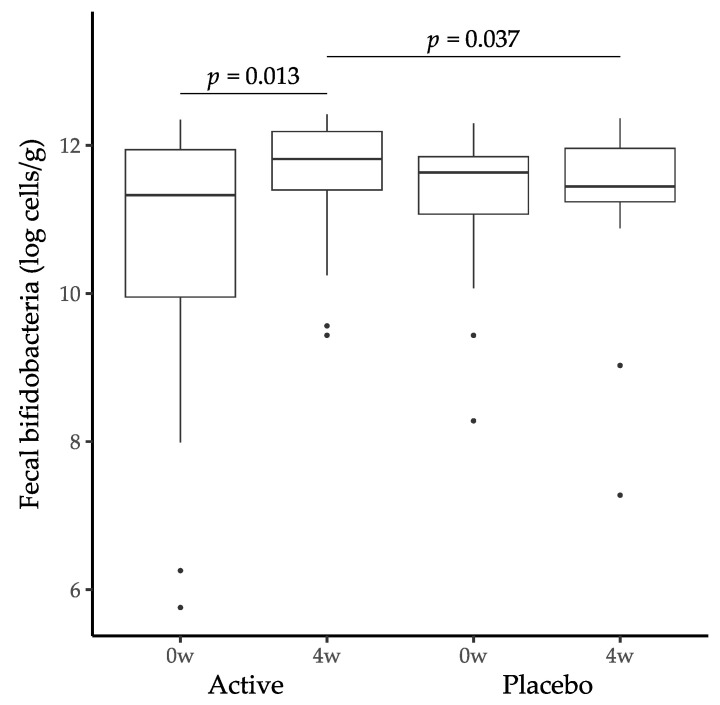
Changes in fecal bifidobacteria in the active (*n* = 20) and placebo (*n* = 20) groups during the study period. Boxplots represent interquartile range (25–75%) with median shown in black. Inter-group differences were analyzed by analysis of covariance with baseline values as covariates. Intra-group differences were analyzed by paired *t*-test.

The quantified number of bifidobacteria in feces was converted into logarithmic values and compared (Figure 2). Inter-group comparison at week 4 revealed that the total number of bifidobacteria was significantly increased in the active group (11.5 ± 0.9 log cells/g feces) compared with the placebo (11.3 ± 1.2 log cells/g feces) (*p* = 0.037 by analysis of covariance with baseline values as covariates). Intra-group comparison revealed a statistically significant increase in the total number of bifidobacteria in the active group at week 4 compared with that at week 0 (10.5 ± 2.0 log cells/g feces) (*p* = 0.013 by paired *t*-test). In contrast, the number of fecal bifidobacteria in the placebo group did not change during the study period (week 0: 11.3 ± 1.0 log cells/g feces).

### 3.5. Fecal Short-Chain Fatty Acids

The fecal concentration of propionic acid in the active group (15.4 ± 6.0 mmol/kg wet feces) at week 4 was statistically lower than in the placebo group (20.9 ± 9.2 mmol/kg wet feces) (*p* = 0.015 by analysis of covariance with baseline values as covariates). In all the items except propionic acid, there were no statistically significant differences between the active group and the placebo during the study period (Table 5).

### 3.6. Anthropometric Parameters

Body weight, BMI, body fat percentage, and muscle mass were measured during the study period (Table 6). There were no statistically significant differences between the active and placebo groups.

## 4. Discussion

We investigated the effect of consuming a dairy drink containing *Bifidobacterium animalis* subsp. *lactis* GCL2505 and inulin on REE in healthy adults. The results showed that the active group had a statistically significantly higher REE score for the primary outcome compared with the placebo group and an increased number of fecal bifidobacteria. Because we have previously shown that GCL2505 and inulin reduce visceral and body fat area, the present results were considered to support earlier findings.

GCL2505 and inulin, which have been shown to inhibit fat accumulation [28], are expected to contribute to the prevention of weight gain and obesity. Obesity is a state of excessive fat accumulation resulting from an imbalance between energy intake and energy expenditure [34]. In other words, continuous intake of GCL2505 and inulin, which has the effect of reducing fat in humans, may cause a rebalancing of energy levels. Thus, the effect of GCL2505 and inulin on REE, which significantly affects the amount of energy a person consumes per day, was verified in this study. The mechanism by which probiotics suppress obesity has been investigated in several studies, and it was reported that the suppressive effect of *Lactobacillus gasseri* SBT2055 on visceral fat accumulation was due to inhibitory effects on the absorption and promotion of lipid excretion in the intestinal tract [35]. Inhibitory effects of *Bifidobacterium breve* B-3 [36] and *Lactobaclillus paracasei* subsp. *paracasei* F199 [37] on fat accumulation in adipose tissue have also been reported. *Lactobacillus gasseri* BNR17 is one of the few bacteria shown to improve energy metabolism. Animal studies have also suggested that the effects of BNR17 on visceral fat accumulation and abdominal circumference reduction depend on increased expression of genes related to fatty acid metabolism [38]. Meanwhile, in the present study, the direct effect of intake of bifidobacteria and dietary fiber on REE was confirmed in clinical trials. REE is strictly determined by summing the metabolic rates of body tissues [39]; however, indirect calorimetry, which is simple, noninvasive, and highly accurate for measurement [40,41], was used in the present study. To date, no studies have demonstrated an ameliorative effect of probiotics on REE, and the reasons for this are not clear. However, for REE to be measured in this study, participants were provided accommodations the day before at the testing facility and were kept in a strictly controlled environment. Given the study design, along with the high ability of GCL2505 and inulin to improve SCFAs levels in the gut, the present study may be the first to demonstrate the effect of probiotics and soluble dietary fiber on REE in a clinical trial. REE (the primary endpoint of this study) was correlated with basal metabolic rate [42], suggesting that the increase in REE may also indicate an increase in the participants’ basal metabolic rate.

It was hypothesized that the increase in REE with the intake of GCL2505 and inulin was achieved by a mechanism of action comprising the following two steps. In Step 1, the intake of GCL2505 and inulin increases bifidobacteria in the gut and increases the levels of SCFAs. In a previous animal study, ingestion of GCL2505 alone contributed to an increase in the number of bifidobacteria in the feces and a concomitant increase in acetic acid levels in the feces and blood [24]. In addition, previous clinical studies reported that GCL2505 and inulin increased the total number of bifidobacteria in feces [43] and that intake of inulin increased SCFAs such as acetic acid via an increase in bifidobacteria [44]. These results support our hypothesis. However, in the present study, we were unable to detect an increase in the levels of SCFAs in the gut as a result of the intervention, and there was no significant difference between the two groups in terms of acetic and butyric acid concentrations in the feces at week 4. Propionate levels in the active group were significantly lower than in the placebo. This discrepancy can be explained by the detectable stability of SCFAs in the gut. The amount of SCFAs present in the feces was known to be highly influenced by stool retention time and other factors [45], with large variations between individuals and between days. Another clinical trial with increased stool collection points and a crossover study design was conducted to confirm the effect of GCL2505 and inulin on the levels of SCFAs in the gut, and the positive impact of the intervention was confirmed (UMIN000050924). In Step 2, the increased levels of SCFAs may activate BAT via the activation of its receptor, G protein-coupled receptor 41 (GPR41), as well as sympathetic nerve stimulation, which, in turn, promotes REE. As mentioned above, previous studies in animals have reported that the administration of acetic acid and butyric acid increases energy expenditure via the activation of brown adipocytes [12,13] and that SCFAs contribute to energy regulation via GPR41 [46]. In addition, SCFAs have been shown to promote the expression of GPR41 and 43 in adipose tissue as well as the differentiation of adipocytes into beige adipocytes, which have the same function as brown adipocytes [47]. In clinical trials, colonic infusion of SCFAs promoted increased lipid oxidation and REE [14]. An observational study in humans revealed a positive correlation between the relative abundance of the genus Bifidobacterium and BAT activity [48], together with the possibility that this effect occurs in an SCFA-dependent manner. These previous studies suggested that SCFAs are likely to have an effect on BAT and REE. Thus, based on the hypotheses thus far, it is speculated that intake of GCL2505 and inulin may increase the concentration of SCFAs in the gut by increasing the total number of bifidobacteria in the gut, thereby stimulating the sympathetic nervous system via GPR41 and activating BAT to improve energy expenditure.

RQ, which is the ratio of carbohydrate to fat oxidation [49], did not change in either the active or placebo groups during the study period. Brooks et al. showed that the energy supply from carbohydrates (glycogen and glucose) increased with increasing exercise intensity, thereby causing the RQ values to change [50]. These previous studies showed that RQ was influenced by environmental factors. Therefore, the stability of the RQ observed in the present study can be attributed to a highly accurate test design that was set up to suppress the influence of environmental factors. The group difference between the active and placebo groups based on the marginal estimated means of REE at week 4 was 84.4 kcal/day (Table 4). It has been reported that the amount of energy deficit required to reduce body weight by 1 kg is around 7400–7700 kcal [51,52]. Based on this concept, the theoretical value of the weight difference between the groups during the current intervention period of 28 days was calculated to be about 0.3 kg. It was considered that the change in values was too small to confirm intervention-induced changes in the body weight and BMI of participants in the active group during this study. However, because the effect on REEs has been confirmed, it is expected that continued intake of GCL2505 and inulin would have a long-term optimizing effect on body weight and BMI. Furthermore, in this study, no aspects of BAT activity were measured, such as body temperature, density of BAT, or cold-induced thermogenesis. In addition, although total bifidobacteria counts were quantified by qPCR, the change in intestinal microbiota was not comprehensively analyzed by 16S amplicon sequence analysis or shotgun metagenomic sequencing. Therefore, further studies are needed to determine the impact of bacteria other than bifidobacteria and whether they influenced the changes in BAT activity or the improvement in REE. Moreover, hypotheses regarding the mechanism may be fully discussed by recruiting a sufficient number of subjects, measuring SCFA accurately while minimizing individual differences and daily variability, and combining this with BAT activity measurements.

Metabolic syndrome, which is the simultaneous development of insulin resistance, obesity, atherosclerosis, and several metabolic diseases represented by dyslipidemia and hypertension, is one of the major public health threats of our time [53]. It is known that the diseases contributing to metabolic syndrome start from obesity, and the overview is sometimes described by the concept of the “metabolic domino effect” [54]. Meanwhile, it has become clear that intestinal bacteria play a pivotal role in maintaining host energy metabolism homeostasis [55,56,57], and probiotics, one of the most promising materials for improving the intestinal environment, are expected to contribute to solving this problem. The ability of probiotics to reach the intestine alive is considered very important for their health benefits to the host. We previously reported that GCL2505 not only reaches the intestinal tract alive after ingestion but also has the characteristic of proliferating in the intestinal tract [21] and has an excellent ability to increase the number of bifidobacteria in the gut [22]. Clinical trials have demonstrated that GCL2505 is effective in reducing visceral fat [58], and animal studies have shown that this effect is due to the high production of SCFAs via the intestinal viability and intestinal proliferation of GCL2505 [59]. In addition, GCL2505 in combination with inulin has been shown in clinical trials to reduce body fat [28], improve vascular endothelial function [26], and improve cognitive function [25]. The effects on cognitive function have been suggested to be due to the anti-inflammatory effects of SCFAs, and further studies are expected. Although a series of research studies have proven the effectiveness of GCL2505 and inulin, the REE improvement effect has been newly revealed in this study. Thus, we can expect that the effects of GCL2505 and inulin on the diseases that constitute metabolic syndrome will contribute to preventing the onset and progression of these diseases in a domino-like fashion, thereby reducing or eliminating the risk of metabolic syndrome.

## 5. Conclusions

The administration of GCL2505 and inulin improved REE by increasing the levels of bifidobacteria in the gut. These results suggest that the intake of GCL2505 and inulin improves the energy balance, which is the underlying cause of obesity. These results might also suggest the underlying mechanism of the anti-obesity effects of GCL2505 and inulin, and this is the first report of the effects of probiotics and dietary fiber on REE.

## Figures and Tables

**Table 1:** Nutritional details of the test drinks.

	Active	Placebo
Energy, kcal/100 g	60.2	47.7
Moisture, g/100 g	82.3	87.0
Protein, g/100 g	2.8	2.8
Fat, g/100 g	0.1	0.1
Carbohydrate, g/100 g	14.9	9.1
Ash, g/100 g	1.1	1.1

The active drink contained 5.0 g of inulin and 1.0 × 10^10^ colony-forming units of GCL2505.

**Table 2:** Participant characteristics at baseline.

	Active Group	Placebo Group	*p*-Value
Age, years	47.75 (11.07)	47.75 (9.68)	1.00
Female, *n* (%)	16 (80.00)	16 (80.00)	1.00
Height, cm	160.18 (6.44)	157.50 (6.45)	0.20
Body weight, kg	68.61 (8.37)	66.86 (6.21)	0.46
Body mass index, kg/m^2^	26.65 (1.47)	26.90 (1.02)	0.53
Systolic blood pressure, mmHg	120.40 (18.19)	123.95 (15.50)	0.51
Diastolic blood pressure, mmHg	79.95 (13.22)	80.90 (10.32)	0.80
White blood cell count, /µL	6130.00 (1475.80)	6000.00 (1069.68)	0.75
Red blood cell count, ×10⁴/µL	470.00 (31.98)	463.90 (38.73)	0.59
Hemoglobin, g/dL	13.77 (1.31)	13.64 (1.26)	0.75
Hematocrit, %	44.18 (3.45)	43.65 (3.29)	0.62
Platelet count, ×10⁴/μL	26.20 (5.01)	29.27 (5.84)	0.08
Total serum protein, g/dL	7.06 (0.37)	7.09 (0.33)	0.79
Aspartate aminotransferase, U/L	21.20 (11.73)	22.35 (12.84)	0.77
Alanine aminotransferase, U/L	22.50 (19.03)	22.95 (17.02)	0.94
Total bilirubin, mg/dL	0.68 (0.33)	0.69 (0.28)	0.96
γ-Glutamyl transpeptidase, U/L	35.05 (50.11)	26.80 (15.07)	0.49
Blood urea nitrogen, mg/dL	12.93 (3.66)	11.29 (1.71)	0.08
Creatinine, mg/dL	0.70 (0.13)	0.68 (0.07)	0.55
Uric acid, mg/dL	5.29 (1.10)	5.64 (1.25)	0.35
Sodium (Na), mEq/L	142.25 (2.12)	141.60 (2.01)	0.33
Chlorine (Cl), mEq/L	102.60 (1.27)	102.25 (2.02)	0.52
Potassium (K), mEq/L	4.15 (0.23)	4.07 (0.28)	0.33
Serum amylase, U/L	80.40 (52.48)	75.50 (26.09)	0.71
Total cholesterol, mg/dL	215.80 (45.89)	225.75 (34.27)	0.44
HDL cholesterol, mg/dL	57.65 (12.52)	55.70 (13.67)	0.64
LDL cholesterol, mg/dL	131.20 (40.90)	139.20 (34.01)	0.51
Triglycerides, mg/dL	118.30 (46.71)	134.20 (59.54)	0.35
Glucose, mg/dL	88.40 (5.78)	93.05 (6.21)	0.02
HbA1c (NGSP), %	5.41 (0.21)	5.47 (0.31)	0.48
Compliance rate of the test sample, % *	99.1 (3.5)	102.7 (7.1)	-

All data are presented as mean (SD). Differences between placebo and active groups were assessed by unpaired *t*-test. * Indicates compliance rate of the test sample, excluding participants who dropped out.

**Table 3:** Changes in dietary composition in the active (*n* = 20) and placebo (*n* = 20) groups during the intervention period.

		0 Week	2 Weeks	4 Weeks
Energy, kcal	Active	2430.02 (1999.86, 2860.17)	2201.98 (1821.45, 2582.50)	2270.44 (1886.14, 2654.74)
	Placebo	2234.22 (1971.28, 2497.17)	2236.50 (1957.50, 2515.50)	1943.19 (1624.80, 2261.58)
	*p*-value	0.45	0.89	0.21
Protein, g	Active	102.14 (82.54, 121.73)	87.80 (70.33, 105.28)	93.64 (75.36, 111.93)
	Placebo	90.81 (78.16, 103.46)	91.12 (77.40, 104.83)	78.38 (64.20, 92.57)
	*p*-value	0.35	0.77	0.20
Fat, g	Active	93.48 (73.39, 113.56)	79.80 (60.79, 98.81)	85.86 (66.31, 105.40)
	Placebo	80.75 (68.49, 93.01)	79.75 (67.23, 92.27)	64.54 (52.08, 77.00)
	*p*-value	0.30	1.00	0.08
Carbohydrate, g	Active	280.39 (234.10, 326.68)	270.57 (233.45, 307.69)	265.11 (230.95, 299.27)
	Placebo	272.41 (242.12, 302.70)	275.72 (238.19, 313.25)	250.93 (211.18, 290.67)
	*p*-value	0.78	0.85	0.60
Dietary fiber, g	Active	15.42 (11.42, 19.42)	14.06 (10.65, 17.48)	14.73 (11.74, 17.71)
	Placebo	15.09 (12.56, 17.62)	14.82 (12.15, 17.48)	14.69 (11.24, 18.13)
	*p*-value	0.89	0.74	0.99

All data are presented as mean (95% CIs). Differences between the placebo and active groups were assessed by unpaired *t*-test.

**Table 4:** Changes in indirect calorimetry in the active (*n* = 20) and placebo (*n* = 20) groups during the intervention period.

		0 Week	2 Weeks	4 Weeks
Resting energy expenditure, kcal/day	Active	1326.8 (1262.9, 1390.7)	1435.9 (1464.2, 1565.5) *	1376.5 (1393.0, 1517.6) *
	Placebo	1325.1 (1257.3, 1392.8)	1345.5 (1362.5, 1463.5)	1303.2 (1308.7, 1433.0)
	Difference between groups	1.8 (−94.4, 97.9)	101.8 (39.2, 164.4)	84.4 (3.2, 165.7)
	*p*-value	0.971	0.002	0.042
Respiratory quotient	Active	0.83 (0.81, 0.85)	0.83 (0.81, 0.85)	0.85 (0.82, 0.87)
	Placebo	0.83 (0.82, 0.84)	0.83 (0.81, 0.84)	0.84 (0.80, 0.86)
	Difference between groups	−0.01 (−0.03, 0.02)	0.00 (−0.02, 0.03)	0.01 (−0.02, 0.05)
	*p*-value	0.649	0.695	0.429
Carbohydrate oxidation amount, mg/min	Active	133.2 (111.57, 154.77)	153.1 (138.6, 178.4)	163.1 (138.7, 198.5)
	Placebo	139.5 (123.95, 155.00)	142.0 (121.9, 162.6)	144.9 (115.0, 175.3)
	Difference between groups	−6.3 (−33.9, 21.3)	16.2 (−9.1, 41.6)	23.4 (−17.2, 64.0)
	*p*-value	0.645	0.202	0.250
Fat oxidation amount, mg/min	Active	82.4 (72.74, 92.02)	85.6 (83.6, 100.9)	76.3 (73.8, 92.4)
	Placebo	79.0 (71.01, 87.08)	80.2 (78.8, 96.0)	74.6 (72.5, 91.1)
	Difference between groups	3.3 (−9.6, 16.3)	4.8 (−6.3, 15.9)	1.3 (−10.8, 13.5)
	*p*-value	0.606	0.382	0.826

All data are presented as mean (95% CIs). Differences between the placebo and active groups were assessed by the mixed-effects model for repeated measures. * *p* < 0.05.

**Table 5:** Changes in fecal SCFA concentrations in the active (*n* = 20) and placebo (*n* = 20) groups during the intervention period.

		0 Week	4 Weeks
Formic acid, mmol/kg wet feces	Active	0.0 (0.0, 0.0)	0.0 (−0.2, 0.2)
	Placebo	0.0 (0.0, 0.0)	0.2 (0.0, 0.5)
	*p*-value	-	0.156
Acetic acid, mmol/kg wet feces	Active	55.6 (43.7, 67.5)	42.4 (34.7, 50.2)
	Placebo	49.6 (40.3, 58.9)	52.9 (45.1, 60.7)
	*p*-value	0.444	0.063
Lactic acid, mmol/kg wet feces	Active	0.4 (−0.4, 1.2)	0.0 (−0.1, 0.1)
	Placebo	0.3 (0.0, 0.6)	0.0 (0.0, 0.1)
	*p*-value	0.759	0.335
Propionic acid, mmol/kg wet feces	Active	19.0 (14.4, 23.6)	15.4 (12.1, 18.5) *
	Placebo	18.6 (13.8, 23.4)	20.9 (17.8, 24.2)
	*p*-value	0.918	0.015
n-Butyric acid, mmol/kg wet feces	Active	9.5 (7.3, 11.7)	7.4 (5.6, 9.2)
	Placebo	7.9 (5.3, 10.6)	8.6 (6.8, 10.4)
	*p*-value	0.377	0.378
iso-Butyric acid, mmol/kg wet feces	Active	0.9 (0.1, 1.7)	0.3 (−0.2, 1.0)
	Placebo	0.0 (0.0, 0.0)	0.6 (0.0, 1.2)
	*p*-value	0.051	0.612
Succinic acid, mmol/kg wet feces	Active	1.0 (0.0, 2.1)	1.4 (−0.1, 2.9)
	Placebo	0.7 (0.2, 1.1)	0.4 (−1.0, 1.9)
	*p*-value	0.547	0.356
n-Valeric acid, mmol/kg wet feces	Active	0.8 (−0.3, 1.9)	0.4 (−0.1, 1.0)
	Placebo	0.3 (−0.3, 0.9)	0.3 (−0.2, 0.9)
	*p*-value	0.449	0.876
iso-Valeric acid, mmol/kg wet feces	Active	1.0 (0.1, 2.0)	0.4 (−0.2, 1.0)
	Placebo	0.0 (0.0, 0.0)	0.6 (−0.1, 1.1)
	*p*-value	0.051	0.808

All data are presented as mean (95% CIs). Differences between the placebo and active groups were assessed by analysis of covariance with baseline values as covariates. * *p* < 0.05.

**Table 6:** Changes in anthropometric parameters in the active (*n* = 20) and placebo (*n* = 20) groups during the intervention period.

		0 Week	2 Weeks	4 Weeks
Body weight, kg	Active	68.6 (64.9, 72.3)	69.0 (67.8, 69.3)	68.9 (67.6, 69.2)
	Placebo	66.9 (64.1, 69.6)	67.1 (67.6, 68.9)	67.1 (67.5, 69.0)
	*p*-value	0.459	0.526	0.726
Body mass index, kg/m^2^	Active	26.6 (26.0, 27.3)	26.8 (26.8, 27.3)	26.7 (26.7, 27.2)
	Placebo	26.9 (26.5, 27.3)	27.0 (26.7, 27.2)	27.0 (26.7, 27.2)
	*p*-value	0.528	0.753	0.965
Body fat rate, %	Active	37.6 (35.1, 40.2)	37.9 (37.0, 38.9)	37.7 (35.5, 40.0)
	Placebo	37.4 (34.6, 40.2)	37.3 (36.5, 38.5)	35.6 (33.6, 38.1)
	*p*-value	0.903	0.291	0.213
Muscle mass, kg	Active	40.5 (37.3, 43.7)	40.5 (40.1, 41.1)	40.6 (39.1, 42.2)
	Placebo	39.7 (36.7, 42.6)	39.9 (40.3, 41.2)	41.1 (40.4, 43.5)
	*p*-value	0.714	0.540	0.239

All data are presented as mean (95% CIs). Differences between the placebo and active group were assessed by the mixed-effects model for repeated measures.

## Data Availability

The datasets generated during the present study and/or analyzed during the current study are available from the responsible author upon reasonable request.

## Supplementary Materials

The following supporting information can be downloaded at: https://www.mdpi.com/article/10.3390/nu16142345/s1, Table S1: CONSORT 2010 checklist.
