# Supplementary material for: Effect of Intake of Bifidobacteria and Dietary Fiber on Resting Energy Expenditure: A Randomized, Placebo-Controlled, Double-Blind, Parallel-Group Comparison Study
Source: Nutrients. 2024 Jul 20;16(14):2345. doi: 10.3390/nu16142345 (PMC11279889; doi:10.3390/nu16142345)
Supplement: Supplementary file 1 [file nutrients-16-02345-s001.zip › Supplementary Table S1.pdf]

Table S1. CONSORT 2010 checklist of information to include when reporting a randomized trial.

Section/Topic	Item No	Checklist item	Reported on page No
**Title and abstract**			
	1a	Identification as a randomised trial in the title	1
	1b	Structured summary of trial design, methods, results, and conclusions (for specific guidance see CONSORT for abstracts)	1
**Introduction**			
Background and objectives	2a	Scientific background and explanation of rationale	1-2
	2b	Specific objectives or hypotheses	2-3
**Methods**			
Trial design	3a	Description of trial design (such as parallel, factorial) including allocation ratio	4-5
	3b	Important changes to methods after trial commencement (such as eligibility criteria), with reasons	N/A
Participants	4a	Eligibility criteria for participants	3
	4b	Settings and locations where the data were collected	4-5
Interventions	5	The interventions for each group with sufficient details to allow replication, including how and when they were actually administered	4-5
Outcomes	6a	Completely defined pre-specified primary and secondary outcome measures, including how and when they were assessed	4-5
	6b	Any changes to trial outcomes after the trial commenced, with reasons	N/A
Sample size	7a	How sample size was determined	4
	7b	When applicable, explanation of any interim analyses and stopping guidelines	N/A
Randomisation:			
Sequence generation	8a	Method used to generate the random allocation sequence	4
	8b	Type of randomisation; details of any restriction (such as blocking and block size)	4
Allocation concealment mechanism	9	Mechanism used to implement the random allocation sequence (such as sequentially numbered containers), describing any steps taken to conceal the sequence until interventions were assigned	4
Implementation	10	Who generated the random allocation sequence, who enrolled participants, and who assigned participants to interventions	4
Blinding	11a	If done, who was blinded after assignment to interventions (for example, participants, care providers, those	4-5

		assessing outcomes) and how	
	11b	If relevant, description of the similarity of interventions	N/A
Statistical methods	12a	Statistical methods used to compare groups for primary and secondary outcomes	7
	12b	Methods for additional analyses, such as subgroup analyses and adjusted analyses	N/A
**Results**			
Participant flow (a diagram is strongly recommended)	13a	For each group, the numbers of participants who were randomly assigned, received intended treatment, and were analysed for the primary outcome	7
	13b	For each group, losses and exclusions after randomisation, together with reasons	7
Recruitment	14a	Dates defining the periods of recruitment and follow-up	5
	14b	Why the trial ended or was stopped	N/A
Baseline data	15	A table showing baseline demographic and clinical characteristics for each group	7-8
Numbers analysed	16	For each group, number of participants (denominator) included in each analysis and whether the analysis was by original assigned groups	7
Outcomes and estimation	17a	For each primary and secondary outcome, results for each group, and the estimated effect size and its precision (such as 95% confidence interval)	9-11
	17b	For binary outcomes, presentation of both absolute and relative effect sizes is recommended	N/A
Ancillary analyses	18	Results of any other analyses performed, including subgroup analyses and adjusted analyses, distinguishing pre-specified from exploratory	N/A
Harms	19	All important harms or unintended effects in each group (for specific guidance see CONSORT for harms)	7
**Discussion**			
Limitations	20	Trial limitations, addressing sources of potential bias, imprecision, and, if relevant, multiplicity of analyses	12-13
Generalisability	21	Generalisability (external validity, applicability) of the trial findings	12-13
Interpretation	22	Interpretation consistent with results, balancing benefits and harms, and considering other relevant evidence	12-13
**Other information**			
Registration	23	Registration number and name of trial registry	14
Protocol	24	Where the full trial protocol can be accessed, if available	5
Funding	25	Sources of funding and other support (such as supply of drugs), role of funders	14
